# Plasmonics for Biosensing

**DOI:** 10.3390/ma12091411

**Published:** 2019-04-30

**Authors:** Xue Han, Kun Liu, Changsen Sun

**Affiliations:** School of Optoelectronic Engineering and Instrumentation Science, Dalian University of Technology, Dalian 116024, China; liukun@dlut.edu.cn (K.L.); suncs@dlut.edu.cn (C.S.)

**Keywords:** plasmonics, resonance modes, biosensing, plasmonic materials, hybrid function, multi-channel sensing, spectroelectrochemistry

## Abstract

Techniques based on plasmonic resonance can provide label-free, signal enhanced, and real-time sensing means for bioparticles and bioprocesses at the molecular level. With the development in nanofabrication and material science, plasmonics based on synthesized nanoparticles and manufactured nano-patterns in thin films have been prosperously explored. In this short review, resonance modes, materials, and hybrid functions by simultaneously using electrical conductivity for plasmonic biosensing techniques are exclusively reviewed for designs containing nanovoids in thin films. This type of plasmonic biosensors provide prominent potential to achieve integrated lab-on-a-chip which is capable of transporting and detecting minute of multiple bio-analytes with extremely high sensitivity, selectivity, multi-channel and dynamic monitoring for the next generation of point-of-care devices.

## 1. Introduction

Along with the fast development in nanofabrication techniques, nano-optics has been boosted and applied in various research and industry areas. Plasmonics is one major branch of nano-optics. Due to its ability to generate nanoscale hot spots which are close to the size of bioparticles, it has been applied in biosensing broadly with enhanced sensitivity for refractive index (RI) changes and enhanced light/matter interactions [1,2,3,4,5]. For example, prostate-specific antigen has been detected with a visual limit of detection (LOD) as low as 0.0093 ng/mL [6]; with the mechanism that glucose oxidase can control the growth of nanoparticles, an inverse sensitivity has been achieved as low as 4 × 10^−20^ M [7]; nanohole array has been used to detect exosomes with the LOD as approximately 670 × 10^−18^ M and potential cancer diagnose without biopsy is possible based on this technique [8], and other applications in bio-interfacial research [9], heavy ions in water [10], foodborne pathogen [11], and drug delivery [12] have been carried out. Some of the plasmonic devices have already been developed into portable manner toward point-of-care (PoC) applications [13,14,15].

Here in this short review, plasmonic biosensing techniques based on nanovoid-type designs are discussed. In Section 2, propagating plasmonic resonance mode based on planar design is mentioned briefly for the introduction on sensing methodologies based on RI changes. For various nanovoid (array) designs, extraordinary transmission effect (EOT), Fabry-Perot (FP) like resonance, and Fano resonance can be excited and used for specific biosensing purposes. In Section 3, plasmonic materials used for biosensing techniques are discussed according to their working frequency range. In Section 4, hybrid sensing techniques by applying the electric conductive function to plasmonic devices are discussed. Plasmonics based on nanoparticles is as important as nanovoid-type plasmonic sensors. These nanoparticles have been used to enhance fluorescence [16,17,18] and Raman spectroscopy [19,20,21,22,23,24,25,26,27,28,29,30,31,32], and to generate heat to introduce convection flows for particle transportation [33]. Currently, hot electron is another hot topic focused on these nanoparticles for plasmonic photocatalytic studies [34,35]. There are comprehensive reviews of plasmonic nanoparticles can be used as reference resources [36,37,38,39,40,41,42,43,44,45].

## 2. Surface Plasmon Resonance

Surface plasmon resonance (SPR) phenomenon happens at the interface of a conductive material and a dielectric medium. Under the resonance condition, the incident light is used to generate a collective charge density wave propagating at this interface and this wave is called surface plasmon polariton (SPP). This phenomenon was first observed by Wood in 1902 [46], and in 1941 Fano proposed a theoretical explanation for Wood’s anomaly by considering a SP as a superficial wave [47]. In 1968 Kretschmann [48] and Otto [49] proposed using a prism coupler to generate SPR respectively, and the first SPR sensor was invented by Liedberg, et al. for gas sensing and antibody-antigen binding events until 1983 [50]. Initially, SPR sensors were mainly based on metal thin films. With the development in nanofabrication techniques, nanostructures have been fabricated in metal thin films and geometric factors have been introduced to control generation conditions of SPPs. In addition, different plasmonic resonance modes and modes coupling have been proposed theoretically and explored experimentally to enhance the nearfield to reduce LOD [51,52]. In this section, film-type sensor is discussed on the purpose to introduce the generation of SPR, plasmonic biosensing mechanisms and interrogation methodologies. Localized SPR (LSPR), EOT effect, FP-like and Fano resonance modes are discussed for nanovoid-type plasmonic sensors.

### 2.1. Film-Type Plasmonic Sensors

#### 2.1.1. SPP Generation

**Figure 1:**
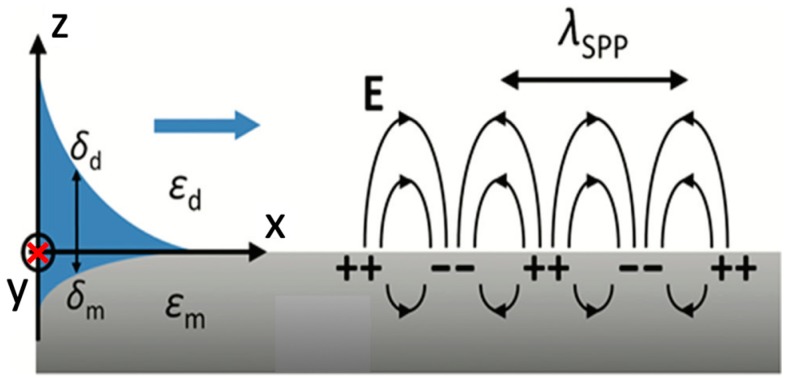
Demonstration of the layer geometry for propagating SPP generation. Adapted from [52], with permission from 2015 © The Royal Society of Chemistry.

Optical thin noble metal films are most used in film-type plasmonic sensors. When Equation (1) is satisfied, a SPP can be generated at the metal/dielectric interface and this type of SPP is also called propagating SPP [50], which is demonstrated as Figure 1.

(1) $$k_{spp,d}\left(\omega \right)=k_{d}{\left(\frac{1}{\varepsilon_{m}\left(\omega \right)}+\frac{1}{\varepsilon_{d}\left(\omega \right)}\right)}^{-1/2}$$

where $k_{d}$ is the wave vector of the incident light in a dielectric medium, $\varepsilon_{m}$ and $\varepsilon_{d}$ is the dielectric constant of the metal material and the dielectric medium respectively, and $k_{spp,d}$ is the wave vector of the SPP which propagates at the interface of this metal and this dielectric medium.

**Figure 2:**
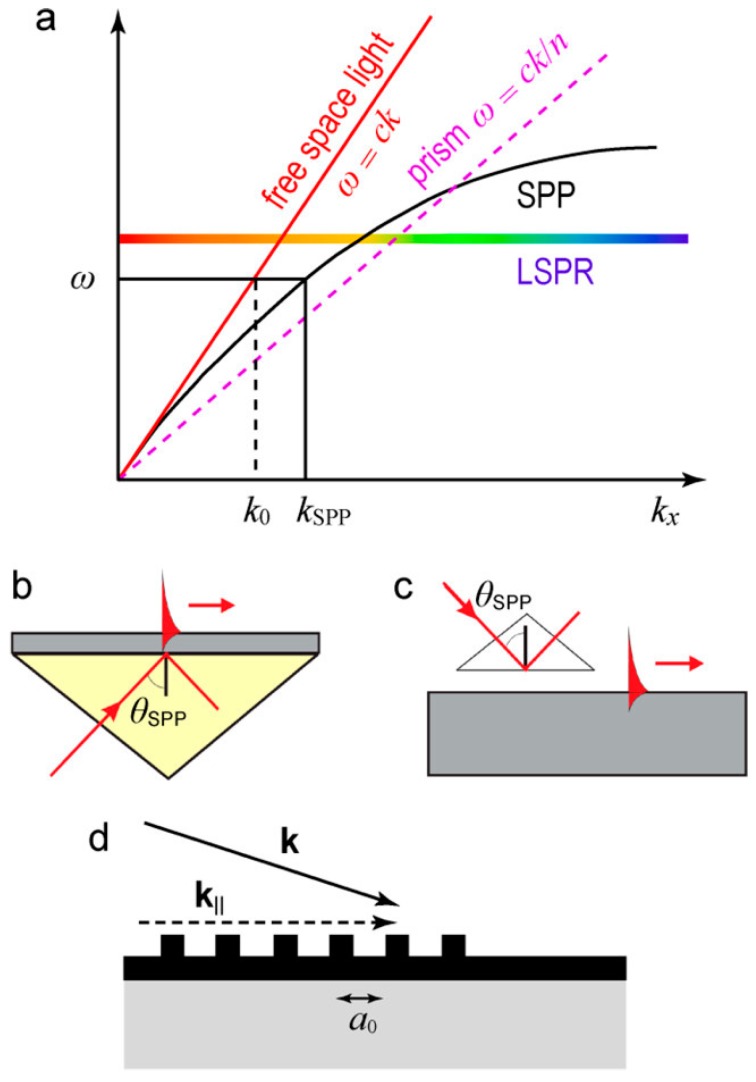
(**a**) Dispersion curves for SPP along metal/free space interface (black curve), light in free space (red curve) and prism material (pink dashed curve); (**b**) Kretschmann; (**c**) Otto and (**d**) grating coupling method. Adapted from [55], with permission © 2017 American Chemical Society.

The demonstrative dispersion curve of SPP ($k_{spp}=k_{spp,0}$) for a metal/free space (dielectric medium, $k_{0}$) interface is plotted as Figure 2a. At the same frequency, $k_{spp}$ is always larger than $k_{0}$. From the free space, simply shining a laser beam at this interface cannot excite a SPP. However, a prism made of glass ($k_{p}>k_{0}$) can be used to match $k_{spp}$ with proper coupling methodologies. In Kretschmann (Figure 2b) and Otto (Figure 2c) coupling methodologies, an evanescent wave from the total internal reflection (TIR) at the metal/prism interface is used to generate SPP at the metal/free space interface. In addition, the evanescent wave of the SPP penetrates into both the metal layer and the dielectric medium with a defined depth $\delta_{m}$ and $\delta_{d}$, as shown in Figure 1. Take a gold/air interface, penetration depths are 328 nm and 28 nm into air and gold separately at 633 nm incident light [53]. These numbers demonstrate that SPP is highly confined at the interface. Gratings also can be used to match the wave vector of a SPP, as shown in Figure 2d. Later coupling techniques based on fibers/waveguides have also been developed [54].

As shown in Equation (1), the dispersion of SPP depends on $\varepsilon_{d}\left(\omega \right)$. When a RI change happens to the dielectric medium that close to the metal surface, which could be a bulk RI change or thin layers added on the surface of the metal, $k_{spp,d}\left(\omega \right)$ is modified to $k_{spp,d{}^{\prime }}\left(\omega \right)$ and the resonance condition/coupling condition is altered. Varied interrogation methodologies can be used to monitor the resonance condition and extract the RI changes of $\varepsilon_{d}\left(\omega \right)$.

#### 2.1.2. Angular Interrogation

**Figure 3:**
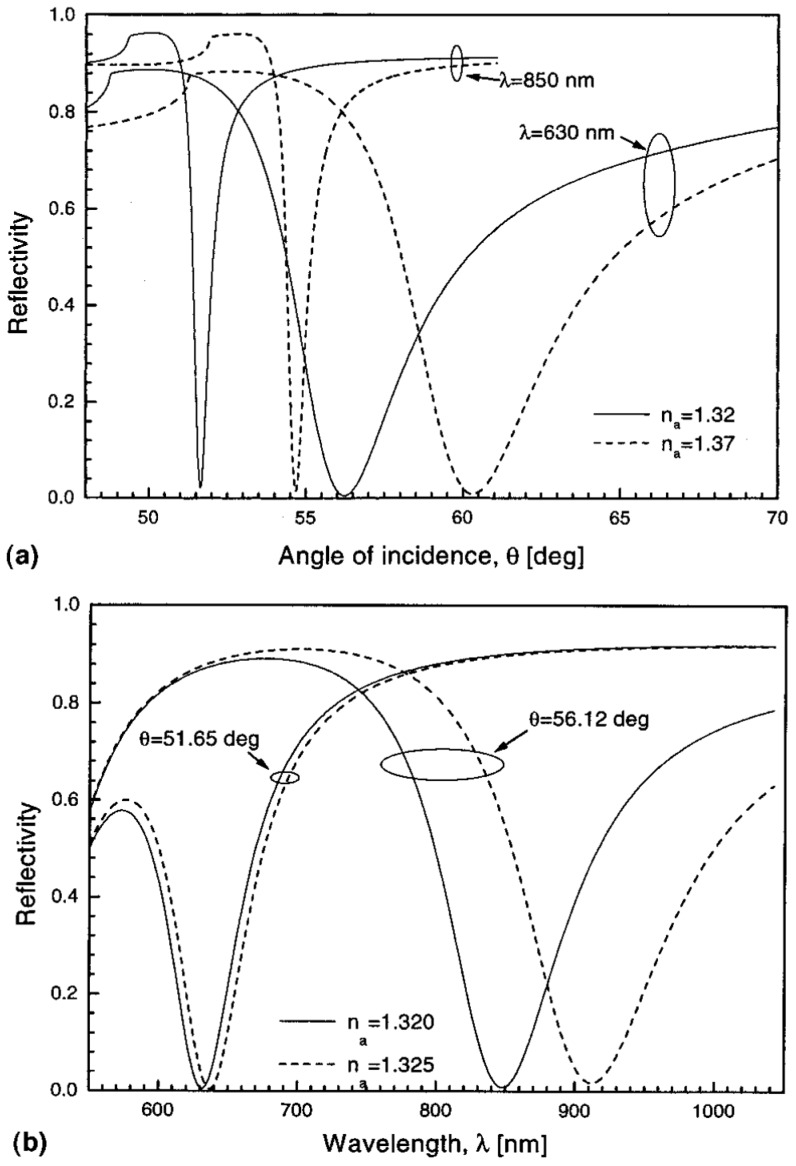
Reflectivity for a system using a prism coupler. (**a**) Angular interrogation. (**b**) Wavelength interrogation. Adapted from [57], with permission © 1999 Elsevier Science S.A.

For an angular interrogation based on a prism coupler, the incident angle should satisfy Equation (2) to generate a SPP and a minimal reflection can be observed at this coupling angle $\theta_{r}$ which is larger than the critical angle of TIR. When there is a RI change happens next to the metal thin film, the coupling angle $\theta_{r}$ is modified to $\theta_{r}^{\prime }$. As shown in Figure 3a, an angular interrogation method is used for bulk RI change from 1.32 to 1.37 at 850 nm and 630 nm incident light.

(2) $$k_{p}\sin\left(\theta_{r}\right)=k_{spp,d}\left(\omega \right)=k_{d}{\left(\frac{1}{\varepsilon_{m}\left(\omega \right)}+\frac{1}{\varepsilon_{d}\left(\omega \right)}\right)}^{-1/2}$$

In an angular interrogation setup, there are two strategies. One is that the incident laser beam is collimated, and a rotation stage is involved to change the incident angle. The incident beam needs to be guaranteed shining on the same spot during a rotation. In this scenario the mechanical vibration is the major factor for the limited sensitivity. The other strategy is that the incident light has a range of angles, and the portion corresponding to the coupling angle is used to generate a SPP and a dark bar is resulted in the reflected angular spectrum. For this scenario, a camera is needed to align incident angles with pixels and the mechanical vibration is deleted from the background noise.

#### 2.1.3. Wavelength Interrogation

In wavelength interrogation, a range of incident wavelengths is used and the incident angle is fixed. At the coupling wavelength, a minimal reflection is observed. As shown in Figure 3b, interrogations using the incident angle at 51.65 degree and 56.12 degree are demonstrated separately to show the shift of the coupling wavelength when the bulk RI changed from 1.320 to 1.325. Compared to an angular interrogation, a wavelength interrogation is more complex in data analysis since a range of refractive indices of the control system (the coupling component, metal thin film and dielectric medium without analyte) is needed. Since no continuously stage rotation is needed in the progress of the measurement, a wavelength interrogation is mechanical stable for sensing applications. Although the mentioned difficulty, spectral SPR sensors has been used to determine the RI dispersion of a variety of biomaterials [56].

The main problem of angular and wavelength interrogation methods is associated with the natural low detection limit of amplitude sensing schemes. This limit is conditioned by the level of noises in measurements and LOD normally is estimated as 10^−5^ to 10^−6^ RI units (RIU) [58].

#### 2.1.4. Phase Interrogation

To increase the detection sensitivity for biomolecules and their interactions at one spot, a phase interrogation was also proposed at the very early stage. In 1976, F. Abeles clearly proposed to use phase interrogation for SPR sensing [59]. To excite SPPs, only p-polarized (TM) incident light can be used for film-type plasmonics, and a dramatic phase shift occurs at the resonance. Interferometry can be used to generate interference pattern if a proper reference beam is selected. However, this methodology only works for film-type SPR when biomolecules are uniformly immobilized on the device surface in a large area which is not practical for biosensing. Another scheme to use this phase-shift character is polarimetry based on an elliposometer. An incident beam containing both s- (TE) and p-polarized components is used, and the ratio between the reflectance of p- and s-polarization is shown as in Equation (3),

(3) $$\frac{r_{p}}{r_{s}}=\tan\Psi e^{i\Delta }=\frac{\left|r_{p}\right|}{\left|r_{s}\right|}e^{i\Delta },\;\Delta =\delta_{p}-\delta_{s}$$

where $r_{p}$ and $r_{s}$ are the complex reflectance for p- and s-polarized components, and Δ is the phase difference (shift) between p- and s-polarized components. When measure $\tan\Psi =\frac{\left|r_{p}\right|}{\left|r_{s}\right|}$ against incident angle or wavelength, a minimal value can be obtained at the resonance condition which is the same as an angular or a wavelength interrogation mentioned previously. For phase interrogation, $e^{i\Delta }$ is retracted from the ellipsometry measurement. Compared to angular or wavelength interrogation, signal/noise ratio can be efficiently increased and improved phase treatments can be applied to obtain the phase shift information. One drawback would be a rotation analyzer or other expensive phase modulation component is needed for the measurement. 

**Figure 4:**
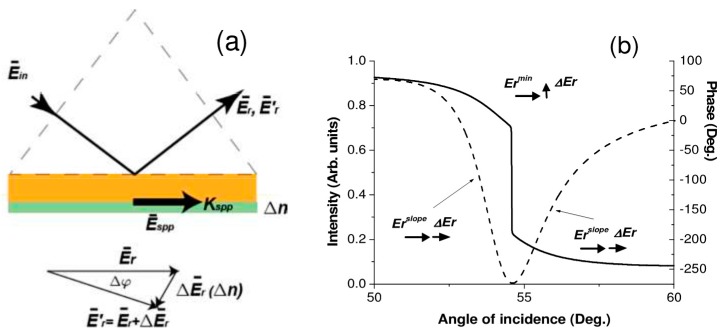
(**a**) SPP generation presented in electric vectors and (**b**) Reflection spectrum of intensity/angle (dashed line) and phase/angle (solid line). Adapted from [60], with permission from © 2009 Optical Society of America.

In Figure 4a, the reflected electric vector $\bar{E_{r}}$ at the initial resonance condition and the one $\bar{E_{r}^{\prime }}$ after a Δn happened next to the metal thin film for an altered resonance condition are presented. Figure 4b compares the reflection intensity and the phase shift against incident angle. A much steeper change around the resonance angle can be observed for the phase curve. This work demonstrates that the LOT of a phase interrogation on the RI change could reach 10^−8^ and even 10^−9^ RIU [60]. Later, novel phase interrogation methodologies have been proposed to further increase the detection sensitivity [61,62] and have been applied in prominent biosensing devices [63,64].

To increase the sensitivity for a RI change introduced by tiny amounts of biomolecules attached on the surface of a SPR sensor, multi-layer designs have also been proposed for propagation SPP to make the resonance curve narrower and deeper [65]. A major design is to add another dielectric waveguide layer on top of the metal thin film and plasmon waveguide resonance (PWR) can be generated. This approach was first proposed by Salamon et al. in 1997 [66]. With both p- and s-polarized incident light, properties of attached biomolecules can be characterized [67,68]. The exceptional narrow line widths of PWR spectra yield enhanced sensitivities which is approximately 20 times better when compared to the conventional SPR sensors on bulk RI changes [69]. Besides this major improvement in sensitivity, the dielectric waveguide coating also provides protection for the metal layer and brings an extra surface immobilization function for various molecules [70]. Interrogation methods mentioned above is based on the RI change introduced by the surface immobilized molecules, hence they are not selective. The selectivity can be introduced by coating a thin layer of specific binding molecules. Along with the interest to improve the sensitivity, introducing selectivity by surface modifications for specific biomolecular interactions and binding events is also under focus for film-type SPR sensors [71].

Film-type SPR sensors have disadvantages in using complex and bulky coupling systems and limited sensitivity due to the poor mechanical and thermal stability. Another major limitation is the narrow range of tunability in the resonance frequency. Determined mainly by the dispersion characteristics of the metal layer and the dielectric bulk medium, there exists a thickness of the metal layer that provides the highest sensitivity. To guarantee a high sensitivity, the resonance frequency only can be tuned in a really narrow range. Practically, a metal thin film with an exact designed thickness cannot be fabricated is the reason that the experimentally determined sensitivity of a film-type SPR sensor is not as good as the theoretical expectation. Based on this extremely narrow tunable range of resonance frequencies, light-matter interactions cannot be used to include intrinsic selectivity naturally into the sensing mechanisms.

### 2.2. Nanovoid-Type Plasmonics

By fabricating nanostructures in conductive thin films, nanoscale geometric factors and/or lattice factors have been introduced to SPPs generation conditions [4,72]. The major advantages of these nanostructures are: (1) they can generate locally enhanced hot spots confined in 3D with a similar size as biological particles which is possible for single biomolecule studies; (2) they has the capability to tune the resonance frequency for a specific light-matter interaction, e.g., Raman spectroscopy, infrared vibrational spectroscopy, or fluorescence; (3) besides various types of resonance modes can be used, modes coupling also can be manipulated to generate much narrower peaks/dips in spectra which can be applied in the detection of minute RI change for a further increased sensitivity [73]; (4) an incident laser beam can be used directly to excite plasmonic resonance without using an external coupling element which reduces the complexity of the entire system. Among versatile designs of nanovoid structures, nanohole [74,75], double nanohole [76] and bowtie aperture structures [77,78] are a few of the most theoretically and experimentally examined ones.

#### 2.2.1. Localized Surface Plasmon Resonance

LSPR mode is mainly used for plasmonics based on nanoparticles. Compared to protruding isolated nanoparticles which generate heat accumulation, nanovoid designs can avoid this issue since the heat dissipates through the rest large area of conductive films. This is beneficial for some biomolecule studies which need to avoid heating effects. For LSPR, the analytical result of the electrical dipole moment for nanoparticles can be used for the nanovoid by exchanging positions of the dielectric constant of the metal nanoparticle and the one of the surrounding medium [53].

Various geometries have been applied to generate LSPR. Single circular nanoholes in optical thin gold films exhibit a distinct tunability in LSPR frequency as the size of the hole changes [79] or as the thickness of the metal film changes [80]. LSPR based on single elongated nanoholes have also been examined and compared to nanodisks [81].

**Figure 5:**
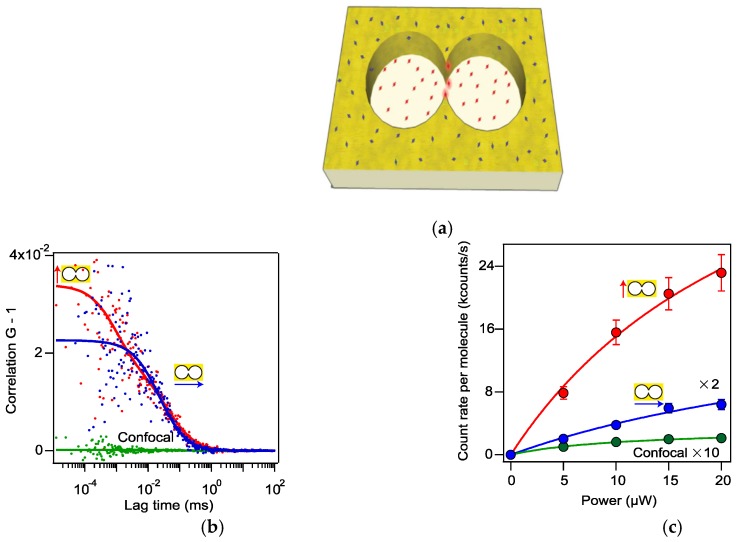
(**a**) Sketch of a double nanohole under an incident light polarized along with the two tips. (**b**) Fluorescence correlation spectroscopies for with double nanohole using incident light polarized parallel (red color) and perpendicular (blue color) to the apex region, and with a confocal microscope (green color). Dotted data is the measurement result and the solid curve is the corresponding fitting result for each case. (**c**) Comparison in brightness of fluorescence among these three cases at different incident power. Adapted from [82], with permission © 2015 Springer Nature Publishing A.G.

To enhance the light-matter interaction, a double nanohole plasmonic design has been used to enhance fluorescence intensity by Regmi et al. [82]. As shown in Figure 5a, LSPR generates nanoscale hot spots at the tips of the double nanohole. With an incident laser beam polarized parallel to the apex region, the locally enhanced nearfield can enhance the fluorescence. Dye molecules near these tips have enhanced fluorescence, and molecules in the double nanohole void are also excited by the incident light without any enhancement, but molecules on top of the gold surface cannot be excited. As shown in Figure 5b, the fluorescence correlation spectroscopy measurements for polarization parallel (red color) and perpendicular to the apex region (blue color), and the fluorescence measurement using a confocal microscope (green color) are plotted to demonstrate the enhancement of fluorescence by using a double nanohole at LSPR. In addition, in Figure 5c, the brightness of fluorescence for these three cases are compared at different incident powers. For clearness, the count was doubled for the perpendicular polarization case and was multiplied by 10 for the confocal measurement. With a double nanohole, the enhancement of fluorescence can be as high as 100-fold.

#### 2.2.2. Magnetic Dipole

As shown in the previous section, an electric dipole can be generated in nanovoids which is a symmetric plasmon mode. When two nanoparticles or nanovoids brought close enough, plasmons hybridization occurs to generate two resonances: one is symmetric that the electric dipolar moments are parallel, and the other one is antisymmetric that the electric dipolar moments are antiparallel. In the second mode, a loop-like current is generated and it can be treated as a magnetic dipolar moment. Currently, most of the research on magnetic dipole is mainly on standing nanostructures [83,84,85], and for modes coupling to generate Fano resonance to have narrow spectra features for RI sensing [86]. 

#### 2.2.3. Toroidal Dipole

**Figure 6:**
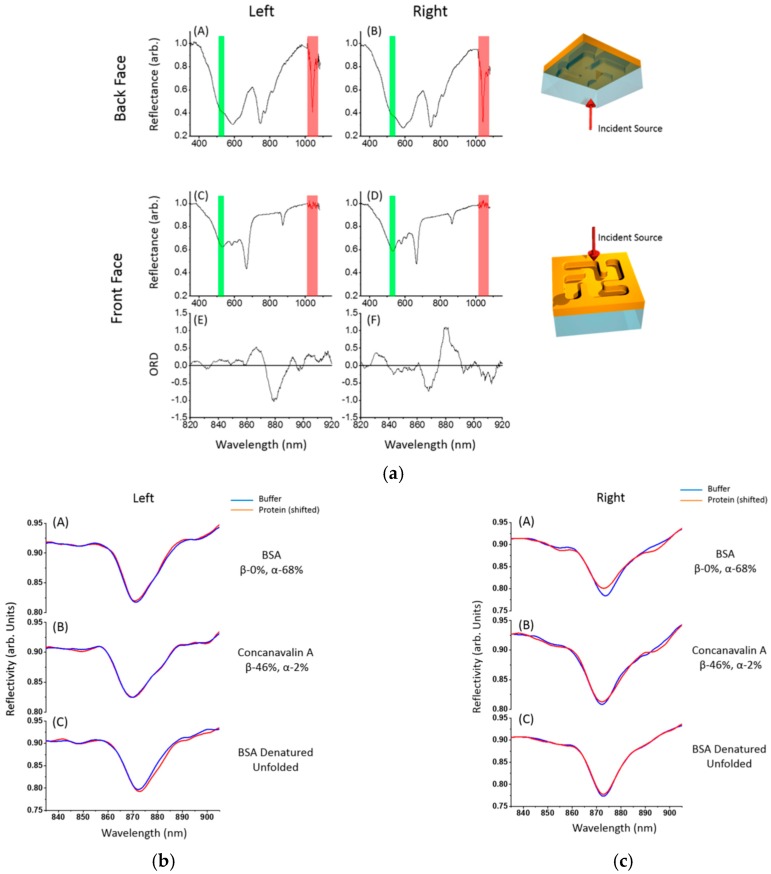
(**a**) Reflectance of left-handed and right-handed structure from back face and front face illuminations, and the ORD is plotted for front face illumination. Red bar represents the laser source, and green bar represents the second harmonic generation (SHG). Reflectivity of 3 types of proteins are plotted for (**b**) left-handed structure and (**c**) right-handed structure. Adapted from [87], with permission © 2016 American Chemical Society.

Most biomolecules are chiral, and chirality can be crucially important, e.g., for a drug molecule. Plasmonic planar chiral metamaterials has been researched to generate chiral electromagnetic (EM) fields to probe chiral molecular structure [87]. As shown in Figure 6a, the reflection spectra from the back-face illumination, the front-face illumination, and its optical rotatory dispersion (ORD) are plotted for the left-handed and right-handed gammadions respectively. Demonstration of both illumination schemes and the sketch of the plasmonic design are also shown. In Figure 6b,c, the measured reflectivity using left-handed and right-handed structures are plotted respectively for 3 types of proteins. These proteins do not show clearly difference from buffer solution when using left-handed structure, while clearly shifts are observed with right-handed structure for natural BSA. Comparing between the natural and denatured BSA protein, it is clearly that this design is sensitive to the second structural information of proteins. The measured differences in the effective refractive indices of chiral samples exposed to left- and right-handed chiral fields generated by these plasmonic nanostructures are found to be up to 10^6^ times greater than those observed in optical polarimetry measurements.

#### 2.2.4. Extraordinary Optical Transmission

When single nanoholes fabricated with an array pattern, EOT phenomenon can be observed easily. The first observation of EOT effect was in 1998 by Ebbesen et al. [88]. They found that sub-micron cylindrical cavities in metallic films displayed highly unusual zero-order transmission spectra at wavelength larger than the array period. Since then theoretical and experimental research have been continuously done on understanding this phenomenon [89] from aspects, e.g., the shape of the nanohole [90] and the width of the nanohole [91].

**Figure 7:**
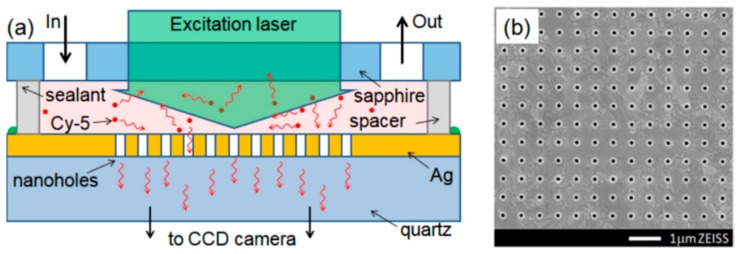
(**a**) Schematic diagram of the sensor, (**b**) SEM image of the nanohole array based on a 100 nm Ag thin film. Adapted from [92], with permission © 2018 Optical Society of America.

For an array of nanoholes, EOT frequency has also been tuned to a fluorescence emission wavelength of the measured Cy-5 molecules to enhance the sensitivity recently by Baburin et al., as shown in Figure 7. Nanoholes with 175 nm diameter are fabricated in 100 nm Ag film with varied periods. In addition, the period which has an EOT peak corresponding to the emission frequency of Cy-5 is selected. Hence the nanohole array is used as an optical filter and the LOD is improved. This application proves the benefit of tuning resonance frequency for a specific light-matter interaction process [92]. For a single nanohole, the EOT effect has also been theoretically described and experimentally achieved [93,94]. With increased mechanical and thermal stabilities, EOT effect from a single nanohole has the potential to be applied in single biomolecule studies.

#### 2.2.5. Fabry-Perot Modes

**Figure 8:**
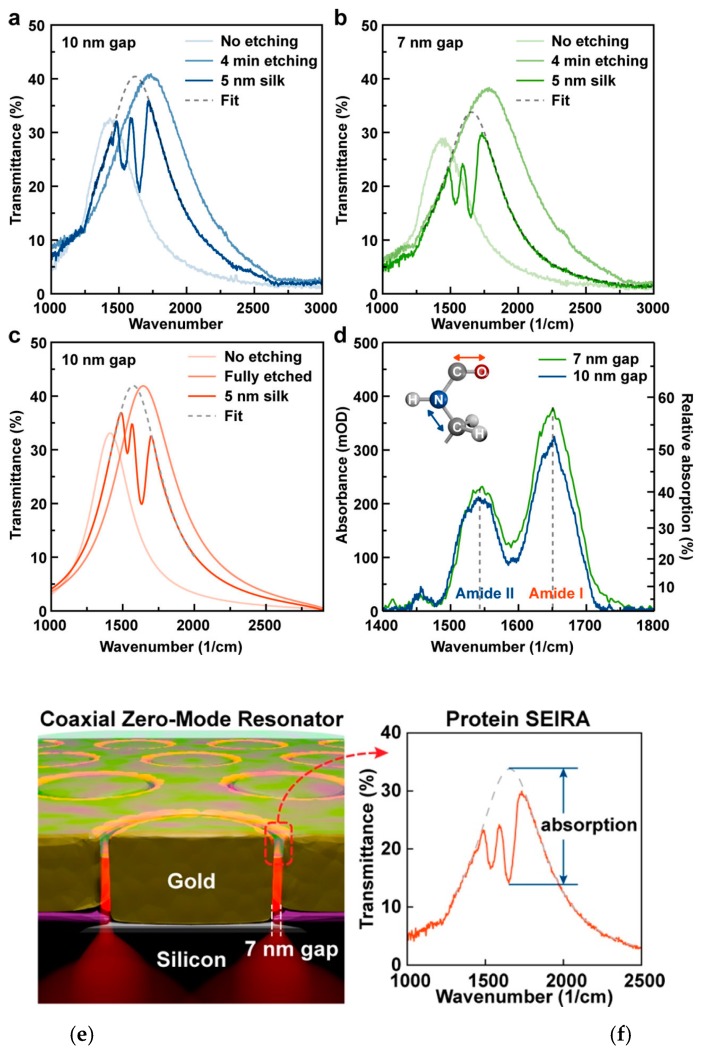
Transmission spectra for no etching, after etching and silk coated conditions for (**a**) 10 nm and (**b**) 7 nm wide nanogap respectively. (**c**) Simulated transmission spectra using 10 nm wide nanogaps under three conditions are plotted for comparison. (**d**) Absorption of 5 nm silk by using 10 nm and 7 nm wide nanogap. (**e**) Schematic presentation of nanogap-based coaxial zero-mode resonator. (**f**) Fitting curve is used in transmission spectra to demonstrate the absorption intensity. Adapted from [99], with permission © 2018 American Chemical Society.

FP like resonance as a guided mode can be generated in a metallic cavity and has a behavior which is well described by the FP formalism. The double nanohole [95], bowtie aperture [96], nanogroove [97] and coaxial nanoring aperture [98] have been explored for FP like resonance. The zeroth-order of FP like resonance has been applied for surface enhanced infrared absorption spectroscopy [99]. As shown in Figure 8, a nanogap with 10 nm (a) and 7 nm (b) width are compared. These nanogaps are originally filled with Al_2_O_3_ as no etching samples. The nanogaps are then etched with H_3_PO_4_, and the transmission spectra are measured after 4 minutes. Since the local RI is reduced by removing the high RI Al_2_O_3_ away, the resonance is observed shifted toward larger wavenumber and the transmission is higher for both nanogaps. Then 5 nm of silk is spin coated on. Dips in transmission spectra are observed due to absorption of silk proteins. A fitting is used to obtain the transmission profile which represents the case without absorption and the fitted resonance frequency is shifted towards the original location. Simulation results are provided for 10 nm wide nanogap for no etching, after etching and silk coating situations, as shown in Figure 8c. The absorption is calculated based on data from (a) and (b) for two nanogap designs, and absorption peaks matching the two infrared vibrational absorption peaks, amide I and II, of silk protein are observed for both designs. The schematic nanogap design is shown in Figure 8e. A 10^4^ to 10^5^ times enhancement on the absorption measurement is shown in Figure 8f.

#### 2.2.6. Fano Resonance

Fano resonance is not the same as Lorentz type of resonance with a symmetric profile. It has an asymmetric profile with a narrow bandwidth. For plasmonics to achieve a Fano resonance, an effective approach is to employ the hybridization of different plasmonic modes [100,101]. Fano resonance has been studied based on multi-bowtie apertures [102] and nanocavity combined with waveguide [103].

**Figure 9:**
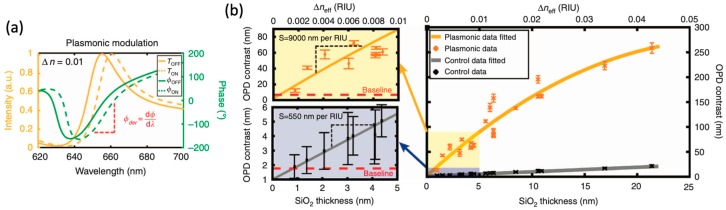
(**a**) Intensity (yellow color) and phase (green color) profiles for 0.01 RIU change. (**b**) OPD contrast comparison between this plasmonic sensor (orange color) and a transparent substrate (grey color) for different thickness of SiO_2_ cover layer. Zoomed in range for thin SiO_2_ is plotted separately for this plasmonic sensor (left top plot) and a transparent substrate (left bottom plot). Adapted from [104], with permission © 2018 Springer Nature Publishing AG.

Fano resonance based on an array of nanoholes has been applied in biosensing with phase interrogation methodology [104]. The Fano resonance is generated by coupling between the surface plasmon mode generated from the grating effect of the nanohole array and the LSPR mode of the single holes. The Fano resonance profile is plotted for both intensity (yellow color) and phase (green color) against incident wavelength, as shown in Figure 9a. In Figure 9b, the optical path difference (OPD) is compared by using a transparent substrate (grey color) and a nanohole array fabricated in Au thin film (orange color) for RI change detection. This change is introduced by changing the thickness of SiO_2_ layer. A thin SiO_2_ layer could represent the situation of a molecular thin film deposited on the surface of the sensor. OPD contrast curves for plasmonic device (left top plot) and a transparent substrate (left bottom plot) are plotted separately. With this plasmonic sensor, the sensitivity for RI detection is as high as 9000 nm/RIU. 

The interrogation methodologies for nanovoid type of plasmonic devices are mainly on wavelength interrogation, while other interrogation methods mentioned previously are all can be used to suit a sensing condition.

## 3. Materials for Plasmonics

As demonstrated in Section 2, the dispersion of the conductive material is the essence to generate plasmonic resonance, and it is also the limitation of the resonance frequency tuning range that a plasmonic device can provide. Natural conductive materials, e.g., metal and graphene, and synthesized materials, e.g., semiconductors and metamaterials, can be used to generate resonance located at different frequency regimes. In this section, materials used for plasmonics are discussed to demonstrate the necessity to select proper materials when a specific range of frequencies is required for biosensing.

### 3.1. Metal

#### 3.1.1. Near Infrared to Long-Wavelength Portion of Visible 

Metals are the most used materials for plasmonics [105,106,107]. At the early stage of the development, research was focused on Ag and Au because of the favorable bulk dielectric properties of these metals.

When the frequency is lower than near infrared (NIR) regime, noble metal can be treated as ideal conductor. Light can be perfectly reflected, and EM wave cannot propagate. In the NIR and visible regime, EM wave penetrates into the metal and loss is increased. SPPs can be generated in this frequency regime [108]. In the ultra violet (UV) regime, metals are transparent, but high energy photons can generate photoelectrons which causes loss [109]. Au and Ag have strong absorption due to the electron bandgap transition. Although Ag has a better dispersion curve in the visible range for plasmonics, it needs protection layers to prevent chemical and mechanical damages which limits its application in biosensing. Due to the chemical and mechanical stability, Au has been more used for plasmonic sensors. In this frequency range, surface plasmonic enhanced infrared vibrational spectroscopy and Raman scattering can be used for biosensing [110,111].

#### 3.1.2. UV to Short-Wavelength Portion of Visible

Motivated by intriguing prospects of combining plasmonic activity with interesting intrinsic targeted materials properties, the plasmonics by using novel metals have received more attention. Currently, research on UV range plasmonics is under focus [112].

Al represents an interesting material both from a fundamental and an applications point of view. It is an abundant and cheap material compared to noble metals. Due to its low price, Al is studied relatively more than the other possible UV plasmonic metals. A reasonably strong interband transition in Al is localized in a narrow energy range around 1.5 eV. Below and above this energy, Al is very much Drude-like. Currently, more research on Al-based plasmonics is focused on optical devices operated in the UV regime, e.g., for optical and plasmonic integrated circuits [113] and color filters [114,115]. In this range of frequencies, surface plasmon enhanced Raman scattering, absorption, fluorescence can be used for biosensing. Al material is not only used for UV plasmonics, but also used for absorbance enhancement in long-wavelength portion of visible regime plasmonics [116,117]. The application of plasmonics based on Al material in biosensing can be expected to increase steeply since related design and fabrication technologies are getting mature [118].

Currently plasmonics based on these UV plasmonic metal materials is in the developing stage. For example, Mg [119,120,121], Ga [122,123], Rh or a few metals combination designs [124] are mainly based on nanoparticle form which are made from chemical synthesis methods. Synthesized nanoparticles have disadvantages in low uniformity, repeatability, and difficulty in manipulation of single nanoparticles for sensing. However, the study of plasmonics using these nanoparticles provides insights for the future nanovoid-type plasmonic devices based on these materials in aspects of geometry design, modes coupling, etc.

### 3.2. Other Materials

Plasmonic biosensors working in infrared regime based on graphene is one the hottest topic recently [125,126,127]. It has been applied as a gas sensor [128] and used for vibrational spectroscopy [129]. Graphene has also been used to combine with other metal materials [130,131]. By adding graphene on top of a thin layer of Au, the sensitivity was improved to 10^−18^ M for single-stranded DNA using phase interrogation [132]. Graphene has also been deposited on top of a nanohole array to enhance the Raman signal 2x10^5^ times [133].

Semiconductor materials and graphene combined with semiconductor are other research directions to control the plasmonic resonance in the mid-infrared regime [134,135,136].

Plasmonics based on magnetic materials is another topic could be interesting for biosensing applications. Nanohole array fabricated in magnetic thin film has been explored for magneto-optical activity introduced by a SPP [137]. Theoretical work on nanohole arrays in Au-Co-Au multi-layer design has been examined for improved sensitivity for RI change [138].

Currently, both novel natural materials and metamaterials are under research for plasmonics [139]. In Table 1, plasmonic sensors based on different materials are listed. Tunable range of the resonance frequency and the sensing mechanism for various analytes are shown to demonstrate the capabilities of current nanovoid-type plasmonic biosensors. Based on specific light-matter interaction mechanisms, materials for the plasmonic biosensor should be selected properly to match the working frequency range to the interaction frequency.

## 4. Hybridization with Electric Conductivity

Due to natural properties of materials used for plasmonic sensors, i.e., electrical conductivity, nanovoid-type plasmonic sensors can hybridize this function for active plasmonics, dielectrophoresis, multi-channel sensing, and electrochemistry which can be beneficial to achieve lab-on-a-chip.

### 4.1. Active Plasmonics

Nanovoid-type plasmonic sensors can have the resonance frequency modified by tuning parameters of the designed geometry before the nanofabrication procedure and this tuning is not dynamic after the sensor is manufactured. An active plasmonic sensor can be beneficial for a biosensing technique to tune the resonance frequency closer to the specific light-matter interaction frequency to enhance detection signal [55].

**Figure 10:**
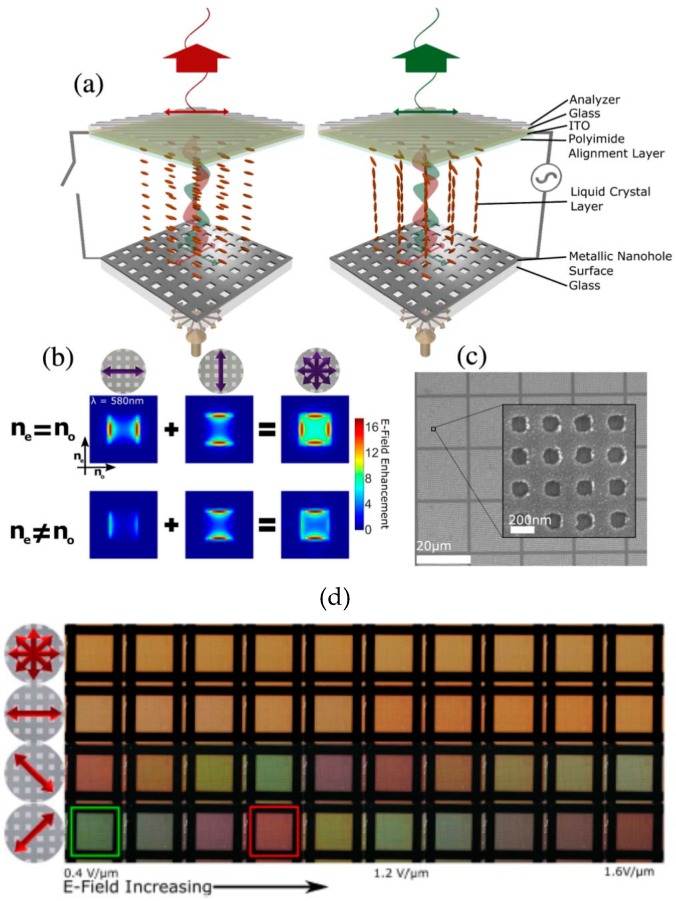
(**a**) Schematic of the LC plasmonic device without and with applied voltage. (**b**) FDTD simulation results of two orthogonal resonance modes. (**c**) SEM image of nanohole array fabricated in Al thin film. (**d**) Appeared color of nanohole arrays under different applied voltages. Adapted from [140], with permission © 2017 Optical Society of America.

Electrical tuning is one of the major methods to have an active plasmonic sensor. With a fixed design, the resonance condition(s) of the sensor can be fine-tuned under an external electrical modulation. There are a few ways to achieve this type of active plasmonic sensors. For example, a layer of liquid crystals (LC) is added on top of a plasmonic nanohole array and by applying an electric field on these crystals the transmission is modified [140]. As shown in Figure 10a, when an electrical modulation is applied between this nanohole array and the upper electrode, the alignment of LC is modified. In Figure 10b, simulated enhanced E-fields are demonstrated for two cases. An image of a nanosquare array fabricated in Al thin film is shown in Figure 10c. When $n_{o}=n_{e}$, the orthogonal modes excited by two linear polarized incident light in a single nanosquare have the same intensity. The light transmits through the nanoholes under these two modes with the same velocity. When $n_{o}\neq n_{e}$, with the same incident wavelength the vertical polarization still excites the vertical resonance mode, while the horizontal polarization cannot excite the horizontal resonance due to the modified RI. When an electrical modulation is applied, the alignment of the LC can be controlled. As a result, the resonance frequencies for these two modes and the appeared color of the whole array are altered, as shown in Figure 10d. This methodology has been proposed for color filter purpose, but the potential to apply this technique in biosensing is obvious.

Research has also been done on electrically tunable imbedded indium tin oxide (ITO) layer to modulate the phase and amplitude of the reflected light respectively which depends on the angle of incidence at the targeted wavelength [141]. This technique can be used for infrared absorption spectroscopy for biosensing. Another possible method is to use graphene as the plasmonic material and apply an electric voltage to alter the carrier density for resonance frequency tuning [129]. Currently, most of active plasmonic devices are targeting optoelectronic applications. Active plasmonic techniques have a huge potential to be used for biosensing to dynamically tune the sensitivity and target different bioparticles or bioprocesses detections.

### 4.2. Particles Transportation

**Figure 11:**
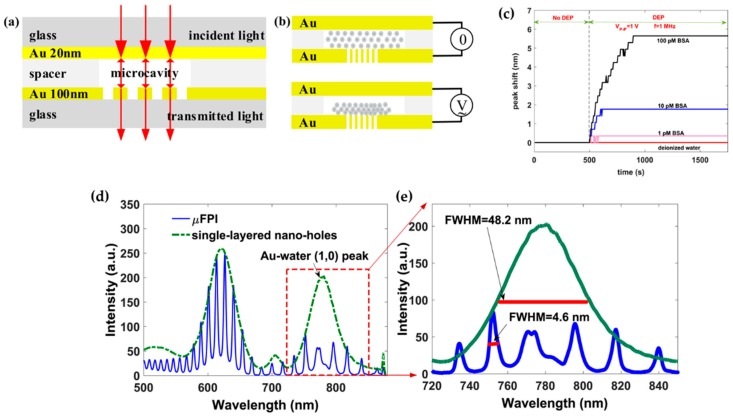
(**a**) Schematic representation of the plasmonic sensing device. (**b**) Conceptual representation of DEP on particle transportation. (**c**) DEP effect on transporting BSA proteins in different concentration solutions. (**d**) Transmission spectrum for the nanohole array with two peaks from the EOT effect (green dash-dot curve) and FP resonances within the transmission profile (blue solid curve). (**e**) Zoomed in curve for the transmission peak in the long wavelength range. Adapted from [143], with permission © 2018 Elsevier B.V.

A nanohole array has been explored to combine with a microfluidic system to transport biomolecules [142]. However, for a nanosize hot spot, bioparticles from a low concentration solution still need long waiting times to get close to the nanoscale sensing area to be detected. To solve this issue, dielectrophoresis (DEP) technique has already been combined with plasmonic devices. A sensor consists of a Au thin film as the top electrode and a Au thin film fabricated with nanohole arrays as the bottom electrode, and it uses DEP to transport biomolecules [143]. In Figure 11a, a nanohole (200 nm in diameter) array in 100 nm thick Au film is used as the lower electrode and the upper Au electrode has a thickness of 20 nm. For plasmonic sensing, EOT effect is applied. In Figure 11b, DEP is demonstrated for the cases without (top figure) and with an electrical potential (bottom figure). The DEP effect on transporting BSA proteins is demonstrated in Figure 11c and different concentrations are tested. In the first 500 s, no electrical potential is applied and no protein is detected. Then DEP effect is activated, and proteins in different concentrations are all detected in a really short time. This result demonstrates an efficient protein transportation using DEP with this design. These two Au layers are also applied as reflection surfaces to form a FP resonance cavity. As shown in Figure 11d, the transmission profile is shown with a dash-dot line with two EOT peaks, and FP resonances can be observed as the blue curve. A zoomed in range of the transmission peak on the long wavelength side of the transmission spectrum is shown in Figure 11e. With the combination of using a micro FP cavity and the EOT effect of a nanohole array, LOD of this design is approximately 0.14 pM by assuming the noise level is 0.05 nm for the wavelength spectrum.

### 4.3. Multi-Channel Sensing

**Figure 12:**
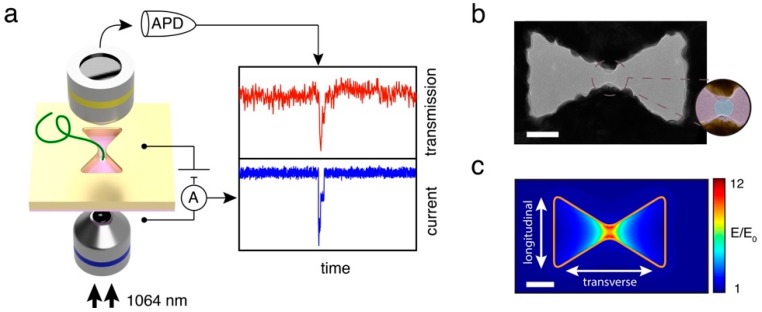
(**a**) Schematic representation of the plasmonic and electrical sensing mechanisms. (**b**) TEM image of the bowtie aperture. (**c**) Simulation result on E-field enhancement. Adapted from [144], with permission © 2018 American Chemical Society.

If a metal thin film fabricated with nanohole arrays is lifted as a membrane suspended in a liquid medium, nanoholes can be used as tunnels for bioparticles to go through. When a particle goes through a tunnel, both the optical and electrical responses of the sensor are modified by this bioparticle occupying the nanovoid. A single bowtie aperture has been explored to transport DNA through, and both optical and electrical signals were examined for its transportation through the hole [144]. As shown in Figure 12a, when a DNA molecule goes through the feed gap of this bowtie aperture, both the optical transmission and electrical current signals has a dip. For the optical signal, the presence of the DNA molecule modified the local RI and hence the resonance frequency is shifted away. In addition, for the electrical signal, the DNA blocks the ion current which causes a drop in the current signal. Figure 12b shows a TEM image of the bowtie aperture fabricated in a 100 nm Au thin film. The across width of the bowtie is 160 nm and the side length is 100 nm. The feed gap is 20 nm. In Figure 12c, the simulated nearfield enhancement is demonstrated over the bowtie aperture. Optical and electrical signals can be used simultaneously to make a sensing technique more comprehensive.

### 4.4. Spectroelectrochemistry

**Figure 13:**
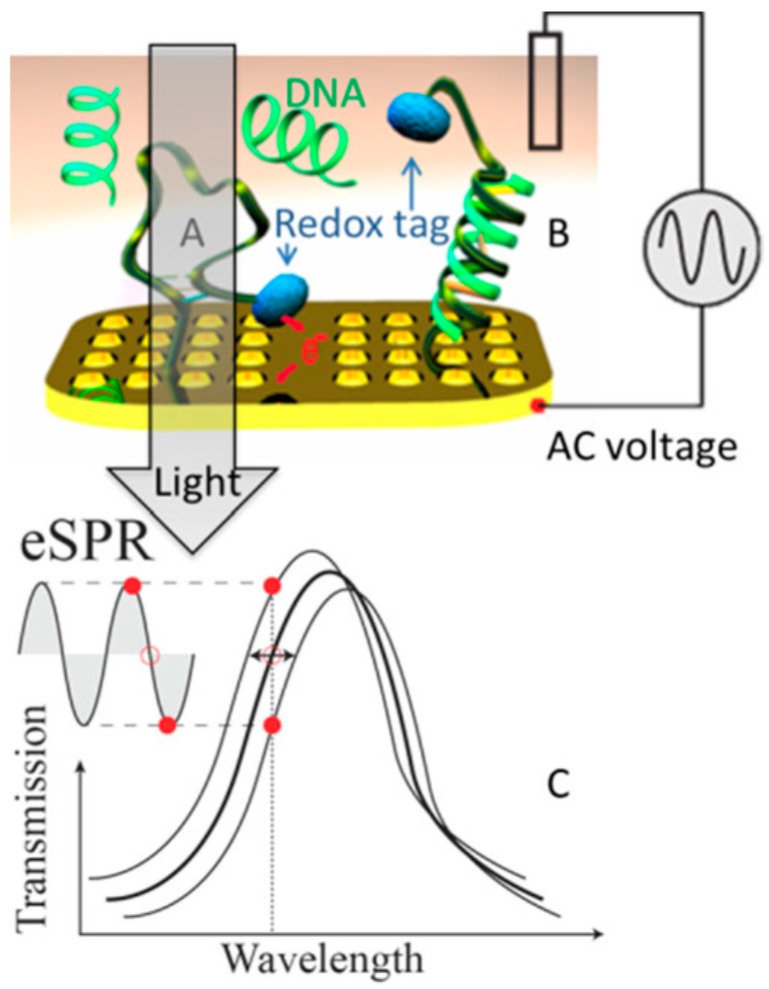
Schematic presentation for electrochemical SPR sensor. (**A**): redox tag attached directly on the surface of the sensor, (**B**): redox tag attached on a DNA, (**C**): transmission for A under a redox reaction. Adapted from [148], with permission © 2015 by WILEY-VCH Verlag GmbH & Co.

As early as the film-type plasmonics, electrochemistry technique was combined with SPR sensing technologies for redox protein studies to avoid the electrical background and further enhance the signal/noise ratio for surface enhanced Raman spectroscopy [145,146]. With the development of plasmonics based on nanostructures, electrochemistry has been coupled to LSPR from nanohole arrays to detect neurotransmitters [147] and DNA-based structure-switching [148]. In this work, nanohole arrays in 100 nm thick Au films with 150 nm diameter and different periods were tested. Three-electrode system was used for the electrochemical experiment. As shown in Figure 13, a redox tag represented in blue color can adsorbed on the sensor surface (A) or attached to a DNA molecule (B). In the first case, the redox tag can have electrochemical reaction. As shown in the bottom Figure 13C, the transmission for this case can be monitored to retract the information of the redox state of the tag. The reduced and oxidized states of the tag have different refractive indices, so the resonance frequency swings back and forth during the redox reaction which appears as an oscillation in the transmission when a fix incident wavelength is used. For the second case, the redox tag is attached to DNA molecule which causes the distance between the redox tag and the sensor surface increased, so the electrochemical reaction is hindered.

As mentioned previously in Section 2, plasmonic sensors based on nanovoid geometries can generate various resonance modes, e.g., toroidal dipole that can be used to detect structural information for biomolecules. These sensors have huge potential to be combined with electrochemistry for redox protein studies to reveal their structural properties dynamically in the process of the redox action.

## 5. Conclusions

Plasmonics, based on materials science and nanostructures, have provided a new playground for researchers to generate desired resonance modes and modes coupling with enhanced nearfield at target frequency which can be used to enhance specific light-matter interactions to further understand the properties of biomolecules and bioprocesses. With its intrinsic electrical property, hybrid functions of active plasmonics, multi-channel sensing, particle transportation and electrochemical study can be added to its nano-optical features for nanovoid-type plasmonic biosensors to be integrated on one chip with high sensitivity, selectivity, simultaneous multiplicity and real time monitoring. In addition, these labs-on-a-chip have huge potential to be used as commercial health instruments for PoC purposes in the near future [149,150].

## Figures and Tables

**Table 1:** Plasmonic biosensors based on different materials.

Materials		Design	Bio-Analyte	Resonance Mode	Experimental RI Sensing	Enhanced Light-Matter Interactions
Metal	Au	Thin-film: 50 nm (2007) [13]	Rocin	Propagating SPP: 635 nm	Angular Interrogation; LOD: ~3.3 × 10^−6^ RIU	N/A
		Nanohole array: hole diameter – 200 nm; array period – 450 nm; thickness of Au – 200 nm (2014) [8]	Exosome (50–100 nm); Selectivity: polyethylene glycol (PEG) +monoclonal antibodies	EOT effect: long-wavelength portion in visible regime	Wavelength Interrogation; LOD: ~670 aM	N/A
		Coaxial nanoring aperture array: depth – 50 nm; inner ring diameter – 170 nm; outer ring diameter – 290 nm; array period – 720 nm; thickness of Au – 250 nm (2018) [72]	poly(allylamine)hydrochloride (PAH, 65 kDa); poly(styrenesulfonate) (PSS, 75 kDa)	Modes coupling of dipolar moments from nanohole and nanodisk; long-wavelength portion in visible regime	Wavelength Interrogation; LOD: ~1.4 × 10^−4^ RIU (estimated)	N/A
		Nanohole array: hole diameter – 200 nm; array period – 600 nm; thickness of Au – 120 nm (2018) [104]	A/G ~ 50 kDa; IgG ~ 150 kDa	EOT effect: long-wavelength portion in visible regime	Phase interrogation; LOD: ~4.5 × 10^−6^ RIU (estimated)	N/A
		Coaxial nanoring aperture array: ring aperture – 7 nm; inner diameter – 710 nm; array period – 720 nm; thickness of Au – 80 nm (2018) [99]	Silk protein: absorbance peaks – 1650 and 1546 cm^−1^	Zeroth-order FP resonance: near infrared regime	N/A	IR absorption enhancement: 10^4^ ∼ 10^5^
	Ag	Nanohole array: hole diameter – 175 nm; array period – 450 nm; thickness of Au – 100 nm (2019) [92]	Cy-5 dye molecules; Excitation wavelength: 628 nm	EOT effect: long-wavelength portion in visible regime	N/A	LOD: Attogram ~ single molecule counting sensors
	Al	Nano bowtie aperture: outline - 450 nm; gap – 30 nm; thickness of Al – 170 nm (2012) [78]	Alexa Fluor 647 molecules; Excitation wavelength: 632.8 nm	Modes coupling of dipolar moments from two arms of the bowtie aperture; visible to near infrared regimes	N/A	Fluorescence enhancement: ~ 12 fold
Graphene		Graphene on 50 nm Au (2015) [132]	24-mer ssDNA (7.3 kDa)	Propagating SPP: 785 nm	Phase interrogation; LOD: ~10^−9^ RIU	N/A
		Ribbon array on gold substrate: 80 nm width (2016) [129]	polyethylene oxide (PED)	Graphene plasmonic resonance: mid-infrared	N/A	Infrared absorption enhancement: ~ 20 fold

## References

[1] Agarwal K., Hwang S., Bartnik A., Buchele N., Mishra A., Cho J.-H. (2018). Small-Scale Biological and Artificial Multidimensional Sensors for 3D Sensing. Small.

[2] Yang X., Sun Z., Low T., Hu H., Guo X., Garcia de Abajo F.J., Avouris P., Dai Q. (2018). Nanomaterial-Based Plasmon-Enhanced Infrared Spectroscopy. Adv. Mater..

[3] Xavier J., Vincent S., Meder F., Vollmer F. (2018). Advances in optoplasmonic sensors—Combining optical nano/microcavities and photonic crystals with plasmonic nanostructures and nanoparticles. Nanophotonics.

[4] Mejia-Salazar J.R., Oliveira O.N. (2018). Plasmonic Biosensing. Chem. Rev..

[5] Taylor A.B., Zijlstra P. (2017). Single-Molecule Plasmon Sensing: Current Status and Future Prospects. ACS Sens..

[6] Guo L., Xu S., Ma X., Qiu B., Lin Z., Chen G. (2016). Dual-color plasmonic enzyme-linked immunosorbent assay based on enzyme-mediated etching of Au nanoparticles. Sci. Rep..

[7] Rodriguez-Lorenzo L., de la Rica R., Alvarez-Puebla R.A., Liz-Marzan L.M., Stevens M.M. (2012). Plasmonic nanosensors with inverse sensitivity by means of enzyme-guided crystal growth. Nat. Mater..

[8] Im H., Shao H., Park Y.I., Peterson V.M., Castro C.M., Weissleder R., Lee H. (2014). Label-free detection and molecular profiling of exosomes with a nano-plasmonic sensor. Nat. Biotechnol..

[9] Jackman J.A., Ferhan A.R., Cho N.J. (2017). Nanoplasmonic sensors for biointerfacial science. Chem. Soc. Rev..

[10] Wang S., Forzani E.S., Tao N. (2007). Detection of heavy metal ions in water by high-resolution surface plasmon resonance spectroscopy combined with anodic stripping voltammetry. Anal. Chem..

[11] Koubova V., Brynda E., Karasova L., Skvor J., Homola J., Dostalek J., Tobiska P., Rosicky J. (2001). Detection of foodborne pathogens using surface plasmon resonance biosensors. Sens. Actuators B Chem..

[12] Kurzatkowska K., Santiago T., Hepel M. (2017). Plasmonic nanocarrier grid-enhanced Raman sensor for studies of anticancer drug delivery. Biosens. Bioelectron..

[13] Feltis B.N., Sexton B.A., Glenn F.L., Best M.J., Wilkins M., Davis T.J. (2008). A hand-held surface plasmon resonance biosensor for the detection of ricin and other biological agents. Biosens. Bioelectron..

[14] Skottrup P.D., Nicolaisen M., Justesen A.F. (2008). Towards on-site pathogen detection using antibody-based sensors. Biosens. Bioelectron..

[15] Ahmadivand A., Gerislioglu B., Manickam P., Kaushik A., Bhansali S., Nair M., Pala N. (2017). Rapid Detection of Infectious Envelope Proteins by Magnetoplasmonic Toroidal Metasensors. ACS Sens..

[16] Bauch M., Dostalek J. (2013). Collective localized surface plasmons for high performance fluorescence biosensing. Opt. Express.

[17] Luan J.Y., Morrisse J.J., Wang Z.Y., Derami H.G., Liu K.K., Cao S.S., Jiang Q.S., Wang C.Z., Kharasch E.D., Naik R.R. (2018). Add-on plasmonic patch as a universal fluorescence enhancer. Light-Sci. Appl..

[18] Fothergill S.M., Joyce C., Xie F. (2018). Metal enhanced fluorescence biosensing: From ultra-violet towards second near-infrared window. Nanoscale.

[19] Luo S.-C., Sivashanmugan K., Liao J.-D., Yao C.-K., Peng H.-C. (2014). Nanofabricated SERS-active substrates for single-molecule to virus detection in vitro: A review. Biosens. Bioelectron..

[20] Feng S., Wang W., Tai I.T., Chen G., Chen R., Zeng H. (2015). Label-free surface-enhanced Raman spectroscopy for detection of colorectal cancer and precursor lesions using blood plasma. Biomed. Opt. Express.

[21] Vo-Dinh T., Wang H.N., Scaffidi J. (2010). Plasmonic nanoprobes for SERS biosensing and bioimaging. J. Biophotonics.

[22] Huang J.A., Zhao Y.Q., Zhang X.J., He L.F., Wong T.L., Chui Y.S., Zhang W.J., Lee S.T. (2013). Ordered Ag/Si nanowires array: Wide-range surface-enhanced Raman spectroscopy for reproducible biomolecule detection. Nano Lett..

[23] Sivapalan S.T., DeVetter B.M., Yang T.K., van Dijk T., Schulmerich M.V., Carney P.S., Bhargava R., Murphy C.J. (2013). Off-Resonance Surface-Enhanced Raman Spectroscopy from Gold Nanorod Suspensions as a Function of Aspect Ratio: Not What We Thought. ACS Nano.

[24] Xu L.-J., Zong C., Zheng X.-S., Hu P., Feng J.-M., Ren B. (2014). Label-Free Detection of Native Proteins by Surface-Enhanced Raman Spectroscopy Using Iodide-Modified Nanoparticles. Anal. Chem..

[25] Wang F., Joshi B.P., Chakrabarty A., Zhang H., Wei Q.-H. (2018). Plasmonic Patch Nanoantennas for Reproducible and High-Sensitivity Chemical Detection with Surface-Enhanced Raman Spectroscopy. ECS Trans..

[26] Gao J., Zhang N., Ji D., Song H., Liu Y., Zhou L., Sun Z., Jornet J.M., Thompson A.C., Collins R.L. (2018). Superabsorbing Metasurfaces with Hybrid Ag-Au Nanostructures for Surface-Enhanced Raman Spectroscopy Sensing of Drugs and Chemicals. Small Methods.

[27] Sivashanmugan K., Liao J.D., Liu B.H., Yao C.K., Luo S.C. (2015). Ag nanoclusters on ZnO nanodome array as hybrid SERS-active substrate for trace detection of malachite green. Sens. Actuators B Chem..

[28] Zrimsek A.B., Wong N.L., Van Duyne R.P. (2016). Single Molecule Surface-Enhanced Raman Spectroscopy: A Critical Analysis of the Bianalyte versus Isotopologue Proof. J. Phys. Chem. C.

[29] Zhang Y., Shen J., Xie Z., Dou X., Min C., Lei T., Liu J., Zhu S., Yuan X. (2017). Dynamic plasmonic nano-traps for single molecule surface-enhanced Raman scattering. Nanoscale.

[30] Darby B.L., Etchegoin P.G., Le Ru E.C. (2014). Single-molecule surface-enhanced Raman spectroscopy with nanowatt excitation. Phys. Chem. Chem. Phys..

[31] Le Ru E.C., Grand J., Sow I., Somerville W.R., Etchegoin P.G., Treguer-Delapierre M., Charron G., Felidj N., Levi G., Aubard J. (2011). A scheme for detecting every single target molecule with surface-enhanced Raman spectroscopy. Nano Lett..

[32] Garcia-Rico E., Alvarez-Puebla R.A., Guerrini L. (2018). Direct surface-enhanced Raman scattering (SERS) spectroscopy of nucleic acids: From fundamental studies to real-life applications. Chem. Soc. Rev..

[33] Yanik A.A., Huang M., Kamohara O., Artar A., Geisbert T.W., Connor J.H., Altug H. (2010). An optofluidic nanoplasmonic biosensor for direct detection of live viruses from biological media. Nano Lett..

[34] Wu N. (2018). Plasmonic metal-semiconductor photocatalysts and photoelectrochemical cells: A review. Nanoscale.

[35] Cushing S.K., Chen C.J., Dong C.L., Kong X.T., Govorov A.O., Liu R.S., Wu N. (2018). Tunable Non-Thermal Distribution of Hot Electrons in a Semiconductor Injected from a Plasmonic Gold Nanostructure. ACS Nano.

[36] Sagle L.B., Ruvuna L.K., Ruemmele J.A., Van Duyne R.P. (2011). Advances in localized surface plasmon resonance spectroscopy biosensing. Nanomedicine.

[37] Petryayeva E., Krull U.J. (2011). Localized surface plasmon resonance: Nanostructures, bioassays and biosensing—A review. Anal. Chim. Acta.

[38] Liu J., He H., Xiao D., Yin S., Ji W., Jiang S., Luo D., Wang B., Liu Y. (2018). Recent Advances of Plasmonic Nanoparticles and their Applications. Materials.

[39] Hanske C., Sanz-Ortiz M.N., Liz-Marzan L.M. (2018). Silica-Coated Plasmonic Metal Nanoparticles in Action. Adv. Mater..

[40] Fan W., Leung M.K. (2016). Recent Development of Plasmonic Resonance-Based Photocatalysis and Photovoltaics for Solar Utilization. Molecules.

[41] Okamoto H., Narushima T., Nishiyama Y., Imura K. (2015). Local optical responses of plasmon resonances visualised by near-field optical imaging. Phys. Chem. Chem. Phys..

[42] Austin L.A., Kang B., El-Sayed M.A. (2015). Probing molecular cell event dynamics at the single-cell level with targeted plasmonic gold nanoparticles: A review. Nano Today.

[43] Zhang S., Geryak R., Geldmeier J., Kim S., Tsukruk V.V. (2017). Synthesis, Assembly, and Applications of Hybrid Nanostructures for Biosensing. Chem. Rev..

[44] Carregal-Romero S., Caballero-Diaz E., Beqa L., Abdelmonem A.M., Ochs M., Huhn D., Suau B.S., Valcarcel M., Parak W.J. (2013). Multiplexed sensing and imaging with colloidal nano- and microparticles. Annu. Rev. Anal. Chem..

[45] Amendola V., Pilot R., Frasconi M., Marago O.M., Iati M.A. (2017). Surface plasmon resonance in gold nanoparticles: A review. J. Phys. Condens. Matter.

[46] Wood R.W. (1902). On a Remarkable Case of Uneven Distribution of Light in a Diffraction Grating Spectrum. Proc. Phys. Soc. Lond..

[47] Fano U. (1941). The Theory of Anomalous Diffraction Gratings and of Quasi-Stationary Waves on Metallic Surfaces (Sommerfeld’s Waves). J. Opt. Soc. Am..

[48] Kretschmann E., Raether H. (1968). Radiative Decay of Non Radiative Surface Plasmons Excited by Light. Z. Naturforsch. A.

[49] Otto A. (1968). Excitation of nonradiative surface plasma waves in silver by the method of frustrated total reflection. Z. Phys. A Hadron. Nucl..

[50] Liedberg B., Nylander C., Lundstrom I. (1983). Surface-Plasmon Resonance for Gas-Detection and Biosensing. Sens. Actuators.

[51] Oh S.H., Altug H. (2018). Performance metrics and enabling technologies for nanoplasmonic biosensors. Nat. Commun..

[52] Smith C.L., Stenger N., Kristensen A., Mortensen N.A., Bozhevolnyi S.I. (2015). Gap and channeled plasmons in tapered grooves: A review. Nanoscale.

[53] Maier S.A. (2007). Plasmonics: Fundamentals and Applications.

[54] Srivastava S.K. (2013). Fiber Optic Plasmonic Sensors: Past, Present and Future. Open Opt. J..

[55] Jiang N., Zhuo X., Wang J. (2018). Active Plasmonics: Principles, Structures, and Applications. Chem. Rev..

[56] Qi Z.M., Wei M.D., Matsuda H., Honma I., Zhou H.S. (2007). Broadband surface plasmon resonance spectroscopy for determination of refractive-index dispersion of dielectric thin films. Appl. Phys. Lett..

[57] Homola J., Koudela I., Yee S.S. (1999). Surface plasmon resonance sensors based on diffraction gratings and prism couplers: Sensitivity comparison. Sens. Actuators B Chem..

[58] Piliarik M., Homola J. (2009). Surface plasmon resonance (SPR) sensors: Approaching their limits?. Opt. Express.

[59] Abelès F. (1976). Surface electromagnetic waves ellipsometry. Surf. Sci..

[60] Kabashin A.V., Patskovsky S., Grigorenko A.N. (2009). Phase and amplitude sensitivities in surface plasmon resonance bio and chemical sensing. Opt. Express.

[61] Shen S., Liu T., Guo J. (1998). Optical phase-shift detection of surface plasmon resonance. Appl. Opt..

[62] Homola J., Yee S.S. (1998). Novel polarization control scheme for spectral surface plasmon resonance sensors. Sens. Actuators B Chem..

[63] Huang Y.H., Ho H.P., Kong S.K., Kabashin A.V. (2012). Phase-sensitive surface plasmon resonance biosensors: Methodology, instrumentation and applications. Ann. Phys..

[64] Deng S., Wang P., Yu X. (2017). Phase-Sensitive Surface Plasmon Resonance Sensors: Recent Progress and Future Prospects. Sensors.

[65] Sreekanth K.V., Alapan Y., ElKabbash M., Ilker E., Hinczewski M., Gurkan U.A., De Luca A., Strangi G. (2016). Extreme sensitivity biosensing platform based on hyperbolic metamaterials. Nat. Mater..

[66] Salamon Z., Macleod H.A., Tollin G. (1997). Coupled plasmon-waveguide resonators: A new spectroscopic tool for probing proteolipid film structure and properties. Biophys. J..

[67] Salamon Z., Tollin G. (2001). Optical anisotropy in lipid bilayer membranes: Coupled plasmon-waveguide resonance measurements of molecular orientation, polarizability, and shape. Biophys. J..

[68] Bahrami F., Maisonneuve M., Meunier M., Aitchison J.S., Mojahedi M. (2013). An improved refractive index sensor based on genetic optimization of plasmon waveguide resonance. Opt. Express.

[69] Byard C.L., Han X., Mendes S.B. (2012). Angle-Multiplexed Waveguide Resonance of High Sensitivity and Its Application to Nanosecond Dynamics of Molecular Assemblies. Anal. Chem..

[70] Salamon Z., Brown M.I., Tollin G. (1999). Plasmon resonance spectroscopy: Probing molecular interactions within membranes. Trends Biochem. Sci..

[71] Linman M.J., Abbas A., Cheng Q. (2010). Interface design and multiplexed analysis with surface plasmon resonance (SPR) spectroscopy and SPR imaging. Analyst.

[72] Liang Y.Z., Li L.X., Lu M.D., Yuan H.Z., Long Z.W., Peng W., Xu T. (2018). Comparative investigation of sensing behaviors between gap and lattice plasmon modes in a metallic nanoring array. Nanoscale.

[73] Liang Y., Zhang H., Zhu W., Agrawal A., Lezec H., Li L., Peng W., Zou Y., Lu Y., Xu T. (2017). Subradiant Dipolar Interactions in Plasmonic Nanoring Resonator Array for Integrated Label-Free Biosensing. ACS Sens..

[74] Dahlin A.B. (2015). Sensing applications based on plasmonic nanopores: The hole story. Analyst.

[75] Eftekhari F., Escobedo C., Ferreira J., Duan X., Girotto E.M., Brolo A.G., Gordon R., Sinton D. (2009). Nanoholes as nanochannels: Flow-through plasmonic sensing. Anal. Chem..

[76] Al Balushi A.A., Zehtabi-Oskuie A., Gordon R. (2013). Observing single protein binding by optical transmission through a double nanohole aperture in a metal film. Biomed. Opt. Express.

[77] Gupta N., Dhawan A. (2018). Bridged-bowtie and cross bridged-bowtie nanohole arrays as SERS substrates with hotspot tunability and multi-wavelength SERS response. Opt. Express.

[78] Lu G.W., Li W.Q., Zhang T.Y., Yue S., Liu J., Hou L., Li Z., Gong Q.H. (2012). Plasmonic-Enhanced Molecular Fluorescence within Isolated Bowtie Nano-Apertures. ACS Nano.

[79] Rindzevicius T., Alaverdyan Y., Sepulveda B., Pakizeh T., Kall M., Hillenbrand R., Aizpurua J., de Abajo F.J.G. (2007). Nanohole plasmons in optically thin gold films. J. Phys. Chem. C.

[80] Park T.H., Mirin N., Lassiter J.B., Nehl C.L., Halas N.J., Nordlander P. (2008). Optical properties of a nanosized hole in a thin metallic film. ACS Nano.

[81] Sepulveda B., Alaverdyan Y., Alegret J., Kall M., Johansson P. (2008). Shape effects in the localized surface plasmon resonance of single nanoholes in thin metal films. Opt. Express.

[82] Regmi R., Al Balushi A.A., Rigneault H., Gordon R., Wenger J. (2015). Nanoscale volume confinement and fluorescence enhancement with double nanohole aperture. Sci. Rep..

[83] Qian Z., Hastings S.P., Li C., Edward B., McGinn C.K., Engheta N., Fakhraai Z., Park S.-J. (2015). Raspberry-like Metamolecules Exhibiting Strong Magnetic Resonances. ACS Nano.

[84] Wu H.W., Han Y.Z., Chen H.J., Zhou Y., Li X.C., Gao J., Sheng Z.Q. (2017). Physical mechanism of order between electric and magnetic dipoles in spoof plasmonic structures. Opt. Lett..

[85] Xi Z., Urbach H.P. (2017). Magnetic Dipole Scattering from Metallic Nanowire for Ultrasensitive Deflection Sensing. Phys. Rev. Lett..

[86] Wang J.Q., Fan C.Z., He J.N., Ding P., Liang E.J., Xue Q.Z. (2013). Double Fano resonances due to interplay of electric and magnetic plasmon modes in planar plasmonic structure with high sensing sensitivity. Opt. Express.

[87] Jack C., Karimullah A.S., Leyman R., Tullius R., Rotello V.M., Cooke G., Gadegaard N., Barron L.D., Kadodwala M. (2016). Biomacromolecular Stereostructure Mediates Mode Hybridization in Chiral Plasmonic Nanostructures. Nano Lett..

[88] Ebbesen T.W., Lezec H.J., Ghaemi H.F., Thio T., Wolff P.A. (1998). Extraordinary optical transmission through sub-wavelength hole arrays. Nature.

[89] García de Abajo F.J. (2007). Colloquium: Light scattering by particle and hole arrays. Rev. Mod. Phys..

[90] Koerkamp K.J., Enoch S., Segerink F.B., van Hulst N.F., Kuipers L. (2004). Strong influence of hole shape on extraordinary transmission through periodic arrays of subwavelength holes. Phys. Rev. Lett..

[91] Van der Molen K.L., Segerink F.B., van Hulst N.F., Kuipers L. (2004). Influence of hole size on the extraordinary transmission through subwavelength hole arrays. Appl. Phys. Lett..

[92] Baburin A.S., Gritchenko A.S., Orlikovsky N.A., Dobronosova A.A., Rodionov I.A., Balykin V.I., Melentiev P.N. (2019). State-of-the-art plasmonic crystals for molecules fluorescence detection. Opt. Mater. Express.

[93] De Abajo F.J.G.I. (2002). Light transmission through a single cylindrical hole in a metallic film. Opt. Express.

[94] Sturman B., Podivilov E., Gorkunov M. (2012). Elementary processes of light transformation for slit structures in real and perfect metals. Photonics Nanostruct. Fundam. Appl..

[95] Chen Y., Kotnala A., Yu L., Zhang J., Gordon R. (2015). Wedge and gap plasmonic resonances in double nanoholes. Opt. Express.

[96] Ibrahim I.A., Mivelle M., Grosjean T., Allegre J.T., Burr G.W., Baida F.I. (2010). Bowtie-shaped nanoaperture: A modal study. Opt. Lett..

[97] Fix B., Jaeck J., Bouchon P., Heron S., Vest B., Haidar R. (2017). High-quality-factor double Fabry-Perot plasmonic nanoresonator. Opt. Lett..

[98] Yoo D., Nguyen N.C., Martin-Moreno L., Mohr D.A., Carretero-Palacios S., Shaver J., Peraire J., Ebbesen T.W., Oh S.H. (2016). High-Throughput Fabrication of Resonant Metamaterials with Ultrasmall Coaxial Apertures via Atomic Layer Lithography. Nano Lett..

[99] Yoo D., Mohr D.A., Vidal-Codina F., John-Herpin A., Jo M., Kim S., Matson J., Caldwell J.D., Jeon H., Nguyen N.-C. (2018). High-Contrast Infrared Absorption Spectroscopy via Mass-Produced Coaxial Zero-Mode Resonators with Sub-10 nm Gaps. Nano Lett..

[100] Chen J.J., Gan F.Y., Wang Y.J., Li G.Z. (2018). Plasmonic Sensing and Modulation Based on Fano Resonances. Adv. Opt. Mater..

[101] Liang Y., Peng W., Li L., Qian S., Wang Q. (2015). Tunable plasmonic resonances based on elliptical annular aperture arrays on conducting substrates for advanced biosensing. Opt. Lett..

[102] Chen Y., Chu J.R., Xu X.F. (2016). Plasmonic Multibowtie Aperture Antenna with Fano Resonance for Nanoscale Spectral Sorting. ACS Photonics.

[103] Lu H., Liu X., Mao D., Wang G. (2012). Plasmonic nanosensor based on Fano resonance in waveguide-coupled resonators. Opt. Lett..

[104] Yesilkoy F., Terborg R.A., Pello J., Belushkin A.A., Jahani Y., Pruneri V., Altug H. (2018). Phase-sensitive plasmonic biosensor using a portable and large field-of-view interferometric microarray imager. Light-Sci. Appl..

[105] McPeak K.M., Jayanti S.V., Kress S.J., Meyer S., Iotti S., Rossinelli A., Norris D.J. (2015). Plasmonic Films Can Easily Be Better: Rules and Recipes. ACS Photonics.

[106] Guay J.M., Cala Lesina A., Cote G., Charron M., Poitras D., Ramunno L., Berini P., Weck A. (2017). Laser-induced plasmonic colours on metals. Nat. Commun..

[107] Barchiesi D., Grosges T. (2014). Fitting the optical constants of gold, silver, chromium, titanium, and aluminum in the visible bandwidth. J. Nanophotonics.

[108] Johnson P.B., Christy R.W. (1972). Optical Constants of the Noble Metals. Phys. Rev. B.

[109] Yakubovsky D.I., Arsenin A.V., Stebunov Y.V., Fedyanin D.Y., Volkov V.S. (2017). Optical constants and structural properties of thin gold films. Opt. Express.

[110] Cheng F., Yang X., Gao J. (2015). Ultrasensitive detection and characterization of molecules with infrared plasmonic metamaterials. Sci. Rep..

[111] Zhong Y.J., Malagari S.D., Hamilton T., Wasserman D. (2015). Review of mid-infrared plasmonic materials. J. Nanophotonics.

[112] Gutierrez Y., de la Osa R.A., Ortiz D., Saiz J.M., Gonzalez F., Moreno F. (2018). Plasmonics in the Ultraviolet with Aluminum, Gallium, Magnesium and Rhodium. Appl. Sci..

[113] Dabos G., Manolis A., Tsiokos D., Ketzaki D., Chatzianagnostou E., Markey L., Rusakov D., Weeber J.C., Dereux A., Giesecke A.L. (2018). Aluminum plasmonic waveguides co-integrated with Si3N4 photonics using CMOS processes. Sci. Rep..

[114] Chen Q., Cumming D.R.S. (2010). High transmission and low color cross-talk plasmonic color filters using triangular-lattice hole arrays in aluminum films. Opt. Express.

[115] Dai P., Wang Y., Zhu X., Shi H., Chen Y., Zhang S., Yang W., Chen Z., Xiao S., Duan H. (2018). Transmissive structural color filters using vertically coupled aluminum nanohole/nanodisk array with a triangular-lattice. Nanotechnology.

[116] Mivelle M., van Zanten T.S., Neumann L., van Hulst N.F., Garcia-Parajo M.F. (2012). Ultrabright bowtie nanoaperture antenna probes studied by single molecule fluorescence. Nano Lett..

[117] White J.S., Veronis G., Yu Z.F., Barnard E.S., Chandran A., Fan S.H., Brongersma M.L. (2009). Extraordinary optical absorption through subwavelength slits. Opt. Lett..

[118] Kunz J.N., Voronine D.V., Lu W., Liege Z., Lee H.W.H., Zhang Z., Scully M.O. (2017). Aluminum plasmonic nanoshielding in ultraviolet inactivation of bacteria. Sci. Rep..

[119] Appusamy K., Blair S., Nahata A., Guruswamy S. (2014). Low-loss magnesium films for plasmonics. Mater. Sci. Eng. B.

[120] Jeong H.H., Mark A.G., Fischer P. (2016). Magnesium plasmonics for UV applications and chiral sensing. Chem. Commun..

[121] Sterl F., Strohfeldt N., Walter R., Walter R., Griessen R., Tittl A., Giessen H. (2015). Magnesium as Novel Material for Active Plasmonics in the Visible Wavelength Range. Nano Lett..

[122] Wu P.C., Losurdo M., Kim T.H., Giangregorio M., Bruno G., Everitt H.O., Brown A.S. (2009). Plasmonic Gallium Nanoparticles on Polar Semiconductors: Interplay between Nanoparticle Wetting, Localized Surface Plasmon Dynamics, and Interface Charge. Langmuir.

[123] Knight M.W., Coenen T., Yang Y., Brenny B.J., Losurdo M., Brown A.S., Everitt H.O., Polman A. (2015). Gallium plasmonics: Deep subwavelength spectroscopic imaging of single and interacting gallium nanoparticles. ACS Nano.

[124] Wu P.C., Losurdo M., Kim T.-H., Garcia-Cueto B., Moreno F., Bruno G., Brown A.S. (2011). Ga–Mg Core–Shell Nanosystem for a Novel Full Color Plasmonics. J. Phys. Chem. C.

[125] Nguyen B.H., Nguyen V.H. (2016). Advances in graphene-based optoelectronics, plasmonics and photonics. Adv. Nat. Sci.-Nanosci. Nanotechnol..

[126] Szunerits S., Boukherroub R. (2018). Graphene-based biosensors. Interface Focus.

[127] Rodrigo D., Limaj O., Janner D., Etezadi D., de Abajo F.J.G., Pruneri V., Altug H. (2015). Mid-infrared plasmonic biosensing with graphene. Science.

[128] Hu H., Yang X., Guo X., Khaliji K., Biswas S.R., Garcia de Abajo F.J., Low T., Sun Z., Dai Q. (2019). Gas identification with graphene plasmons. Nat. Commun..

[129] Hu H., Yang X., Zhai F., Hu D., Liu R., Liu K., Sun Z., Dai Q. (2016). Far-field nanoscale infrared spectroscopy of vibrational fingerprints of molecules with graphene plasmons. Nat. Commun..

[130] Losurdo M., Yi C., Suvorova A., Rubanov S., Kim T.H., Giangregorio M.M., Jiao W., Bergmair I., Bruno G., Brown A.S. (2014). Demonstrating the capability of the high-performance plasmonic gallium-graphene couple. ACS Nano.

[131] Pau J.L., García-Marín A., Hernández M.J., Lorenzo E., Piqueras J. (2016). Optical biosensing platforms based on Ga-graphene plasmonic structures on Cu, quartz and SiO2/Si substrates. Phys. Status Solidi (b).

[132] Zeng S., Sreekanth K.V., Shang J., Yu T., Chen C.K., Yin F., Baillargeat D., Coquet P., Ho H.P., Kabashin A.V. (2015). Graphene-Gold Metasurface Architectures for Ultrasensitive Plasmonic Biosensing. Adv. Mater..

[133] Mahigir A., Chang T.W., Behnam A., Liu G.L., Gartia M.R., Veronis G. (2017). Plasmonic nanohole array for enhancing the SERS signal of a single layer of graphene in water. Sci. Rep..

[134] Barho F.B., Gonzalez-Posada F., Milla-Rodrigo M.J., Bomers M., Cerutti L., Taliercio T. (2016). All-semiconductor plasmonic gratings for biosensing applications in the mid-infrared spectral range. Opt. Express.

[135] Agarwal A., Vitiello M.S., Viti L., Cupolillo A., Politano A. (2018). Plasmonics with two-dimensional semiconductors: From basic research to technological applications. Nanoscale.

[136] Kim J., Choudhury S., DeVault C., Zhao Y., Kildishev A.V., Shalaev V.M., Alù A., Boltasseva A. (2016). Controlling the Polarization State of Light with Plasmonic Metal Oxide Metasurface. ACS Nano.

[137] Maccaferri N., Inchausti X., García-Martín A., Cuevas J.C., Tripathy D., Adeyeye A.O., Vavassori P. (2015). Resonant Enhancement of Magneto-Optical Activity Induced by Surface Plasmon Polariton Modes Coupling in 2D Magnetoplasmonic Crystals. ACS Photonics.

[138] Caballero B., García-Martín A., Cuevas J.C. (2016). Hybrid Magnetoplasmonic Crystals Boost the Performance of Nanohole Arrays as Plasmonic Sensors. ACS Photonics.

[139] Naik G.V., Shalaev V.M., Boltasseva A. (2013). Alternative Plasmonic Materials: Beyond Gold and Silver. Adv. Mater..

[140] Bartholomew R., Williams C., Khan A., Bowman R., Wilkinson T. (2017). Plasmonic nanohole electrodes for active color tunable liquid crystal transmissive pixels. Opt. Lett..

[141] Lee Y., Kim S.J., Yun J.G., Kim C., Lee S.Y., Lee B. (2018). Electrically tunable multifunctional metasurface for integrating phase and amplitude modulation based on hyperbolic metamaterial substrate. Opt. Express.

[142] Abbas A., Linman M.J., Cheng Q. (2011). New trends in instrumental design for surface plasmon resonance-based biosensors. Biosens. Bioelectron..

[143] Tu L., Huang L., Wang W. (2019). A novel micromachined Fabry-Perot interferometer integrating nano-holes and dielectrophoresis for enhanced biochemical sensing. Biosens. Bioelectron..

[144] Verschueren D.V., Pud S., Shi X., De Angelis L., Kuipers L., Dekker C. (2018). Label-Free Optical Detection of DNA Translocations Through Plasmonic Nanopores. ACS Nano.

[145] Wu D.Y., Li J.F., Ren B., Tian Z.Q. (2008). Electrochemical surface-enhanced Raman spectroscopy of nanostructures. Chem. Soc. Rev..

[146] Liao W.C., Annie Ho J.A. (2014). Improved activity of immobilized antibody by paratope orientation controller: Probing paratope orientation by electrochemical strategy and surface plasmon resonance spectroscopy. Biosens. Bioelectron..

[147] Li N., Lu Y., Li S., Zhang Q., Wu J., Jiang J., Liu G.L., Liu Q. (2017). Monitoring the electrochemical responses of neurotransmitters through localized surface plasmon resonance using nanohole array. Biosens. Bioelectron..

[148] Patskovsky S., Dallaire A.-M., Blanchard-Dionne A.-P., Vallée-Bélisle A., Meunier M. (2015). Electrochemical structure-switching sensing using nanoplasmonic devices. Ann. Phys..

[149] Lopez G.A., Estevez M.C., Soler M., Lechuga L.M. (2017). Recent advances in nanoplasmonic biosensors: Applications and lab-on-a-chip integration. Nanophotonics.

[150] Belkin M., Chao S.H., Jonsson M.P., Dekker C., Aksimentiev A. (2015). Plasmonic Nanopores for Trapping, Controlling Displacement, and Sequencing of DNA. ACS Nano.

